# Treatment-related adverse events associated with HER2-Targeted antibody-drug conjugates in clinical trials: a systematic review and meta-analysis

**DOI:** 10.1016/j.eclinm.2022.101795

**Published:** 2022-12-27

**Authors:** Zhiwen Fu, Jinmei Liu, Shijun Li, Chen Shi, Yu Zhang

**Affiliations:** a Department of Pharmacy, Union Hospital, Tongji Medical College, Huazhong University of Science and Technology, China; b Hubei Province Clinical Research Centre for Precision Medicine for Critical Illness, China

**Keywords:** Adverse events, HER2-Targeted ADCs, Clinical trials, Systematic review, Bayesian meta-analysis

## Abstract

**Background:**

Given the increasing use of HER2-targeted antibody–drug conjugates (ADCs) worldwide, the summary of toxicity incidence and profiles of these drugs is crucial to provide reference for clinical application. This meta-analysis aimed to estimate the mean incidences of treatment-related adverse events of HER2-targeted ADCs and to investigate the differences between different drugs and cancer types.

**Methods:**

We performed a systematic search of literature in PubMed, Embase, Web of Science, and Scopus databases from inception to February 1, 2022 and the last search was updated to August 1, 2022. Published prospective clinical trials on single-agent of the US Food and Drug Administration approved HER2-targeted ADCs with available count data regarding treatment-related adverse events were included. The primary outcomes were pooled incidences of treatment-related adverse events and differences between different drugs and cancer types. The data synthesis was performed using a Bayesian hierarchical modelling method and the protocol was registered in PROSPERO (CRD42022331627).

**Findings:**

A total of 39 studies (37 trials) involving 7688 patients across five cancer types were included in the final analysis. On pooling the data using Bayesian hierarchical modelling, the overall mean incidence of all-grade adverse events, high-grade adverse events, serious adverse events, and adverse events that resulted in drug discontinuation were 98.29% (95% CrI, 97.33%–99.07%, τ = 1.49), 47.88% (95% CrI, 42.74%–53.17%, τ = 0.37), 19.45% (95% CrI, 15.70%–23.67%, τ = 0.55), and 10.52% (95% CrI, 8.03%–13.21%, τ = 0.56), respectively. The most common all-grade adverse events were nausea (41.57%; 95% CrI, 40.46%–42.64%, τ = 0.81), fatigue (35.86%; 95% CrI, 34.85%–36.96%, τ = 0.65), and decreased appetite (28.84%; 95% CrI, 22.93%–36.87%, τ = 0.76). The most common high-grade adverse events were thrombocytopenia (8.37%; 95% CrI, 7.75%–9.07%, τ = 0.71), anaemia (6.49%; 95% CrI, 5.86%–7.11%, τ = 1.06), and neutropenia (6.42%; 95% CrI, 5.76%–7.04%, τ = 1.21). We found no difference in the mean incidences of adverse events among different cancer types, as well as different dosing regimens. However, trastuzumab deruxtecan (T-DXd) appeared to have higher mean incidences of adverse events compared with trastuzumab emtansine (T-DM1), especially for the higher dose of T-DXd (6.4 mg/kg Q3W).

**Interpretation:**

The incidences of adverse events between two HER2-targeted ADCs were similar in different cancer types, but different HER2-targeted ADCs appeared to have different mean incidences of adverse events. The comprehensive summary of the adverse events of HER2-targeted ADCs is critical for clinicians caring for patients with cancer receiving HER2-targeted ADCs therapy.

**Funding:**

10.13039/100014717The National Natural Science Foundation of China (Grant No. 82073402) and Key R&D Plan of Hubei Province, China (No.2020BCA060) funded this study.

**Research in context**

**Evidence before this study**

HER2-targeted antibody-drug conjugates (ADCs) play important roles in targeted cancer therapy and have been increasingly used worldwide. Concerns have been raised about its safety issues because these emerging drugs might cause some undesirable toxicities, there is an urgent need to comprehensively investigate the toxicity profiles of HER2-targeted ADCs. We performed preliminary searches in PubMed, Embase, Scopus and Web of Science databases from inception to January 25, 2022 with the search terms “trastuzumab deruxtecan (T-DXd)”, “trastuzumab emtansine (T-DM1)”, “adverse event”, and “clinical trials”. We found several prospective clinical trials evaluating the efficacy of HER2-targeted ADCs in patients with cancer which also involved the investigation on adverse events, however, there is no systematic review and meta-analysis for comprehensive summary of treatment-related adverse events associated with HER2-targeted ADCs except the single drug T-DM1. Therefore, current available studies are insufficient to provide the real incidences and to comprehensively understand the adverse events associated with HER2-targeted ADCs.

**Added value of this study**

To the best of our knowledge, this is the first and the most comprehensive meta-analysis of treatment-related adverse events for HER2-targeted ADCs in cancer patients. The overall mean incidence of all-grade adverse events, high-grade adverse events, serious adverse events, and adverse events that resulted in drug discontinuation were 98.29% (95% CrI, 97.33%–99.07%), 47.88% (95% CrI, 42.74%–53.17%), 19.45% (95% CrI, 15.70%–23.67%), and 10.52% (95% CrI, 8.03%–13.21%), respectively. The mean incidences of treatment-related adverse events were similar across different cancer types but varied between different HER2-targeted ADCs.

**Implications of all the available evidence**

The incidences of adverse events between two HER2-targeted ADCs were similar in different cancer types, but different HER2-targeted ADCs appeared to have different mean incidences of adverse events. A comprehensive summary of treatment-related adverse events associated with HER2-targeted ADCs in clinical trials is crucial to provide reference for clinical practice.

## Introduction

Since the successful approval of trastuzumab emtansine (T-DM1) in 2013, antibody-drug conjugates (ADCs) targeting the human epidermal growth factor receptor 2 (HER2) have represented one of the important HER2-targeted therapies.^1^ HER2-targeted ADC could deliver the cytotoxic drug into tumour cells through the targeting property of the antibody to achieve precise and effective killing of tumour cells. HER2 is currently the number one target for ADC drug development worldwide.^2^ To date, two HER2-targeted ADCs (T-DM1 and trastuzumab deruxtecan) have been approved by the US Food and Drug Administration (FDA) and over 60 HER2-targeted ADC candidates including those with novel HER2-targeting bispecific antibodies are being tested in different stages of clinical trials.^3^^,^^4^

Benefiting from the ingenious molecular design that accurately brings cytotoxic drugs into cancer cells, T-DM1 and trastuzumab deruxtecan (T-DXd) have been gradually expanded indications with excellent efficacy.^5^, ^6^, ^7^ However, the antibody-independent internalization and the premature release of payload in circulation have been reported to cause undesirable toxicities in HER2-targeted ADCs.^8^ Organ specific treatment-related adverse events are pneumonitis, haematotoxicity, cardiotoxicity, and hepatotoxicity as well as more general adverse events, including nausea, fatigue, diarrhoea and decreased appetite.^9^^,^^10^ Toxicity profiles are important factors to consider when obtaining informed consent from patients and a clear understanding of HER2-ADCs associated adverse events is necessary to assess the benefit-risk ratio and enable their proper management. Therefore, given the increasing use of HER2-targeted ADCs worldwide, to investigate the incidences and profiles of treatment-related adverse events associated with approved HER2-ADCs would be crucial to provide reference for clinicians to prescribe HER2-ADCs.

In recent decades, multiple clinical trials have been performed to investigate the efficacy and safety profile of HER2-targeted ADCs.^11^, ^12^, ^13^, ^14^, ^15^, ^16^, ^17^, ^18^ The treatment-related adverse events reported in these trials were generally based on the same criteria using the National Cancer Institute Common Terminology Criteria for Adverse Events (CTCAE), which provide the suitable resources to systematically analyse the toxicologic profile of HER2-targeted ADCs. Although several previous publications have reported the meta-analysis result of T-DM1 that involved the treatment-related adverse events, there is lack of T-DXd and they were performed with the few number of included studies or were focused on the efficacy evaluation and some certain adverse events, such as thrombocytopenia and hepatotoxicity.^19^, ^20^, ^21^ Current available studies are insufficient to provide the real incidences and to comprehensively understand the adverse events associated with HER2-targeted ADCs.

We herein performed a systematic review and meta-analysis of the toxicity profile of HER2-targeted ADCs. Using a Bayesian hierarchical modelling method, the incidences and profiles of the treatment-related adverse events of HER2-targeted ADCs were investigated through a quantitative combination of data in published clinical trials. To better provide a reference for clinicians, the main causes of death related to HER2-targeted ADCs and the differences between different drugs and cancer types were also identified.

## Methods

### Search strategy and selection criteria

This systematic review and meta-analysis were conducted in accordance with Preferred Reporting Items for Systematic Reviews and Meta-Analyses (PRISMA) guidelines.^22^ The protocol was registered in PROSPERO (No. CRD42022331627). We performed a systematic search of literature regarding treatment-related adverse events of HER2-targeted ADCs in PubMed, Embase, Web of Science, and Scopus databases from inception to February 1, 2022. The full search strategy was detailed in Supplementary file 1 and the last search was updated to August 1, 2022. The additional relevant trials were supplemented by the manual screening of reference lists from review papers. Trials meeting all of the following criteria were included for analysis: (1) published prospective clinical trials, (2) participants were adults (≥18 years of age) who received a single agent of the FDA-approved HER2-targeted ADC, and (3) available count data regarding treatment-related adverse events. To allow for comparability between trials, only trials in which adverse events were reported based on the CTCAE guideline were included. Studies not matching the selection criteria and studies in which the number of patients in the HER2-targeted ADC group was less than ten were excluded. Whenever publications report the same trial, we included the most recent one. All the trials to be included were screened and reviewed independently by two authors (SJ Li and JM Liu) of our team through abstract and full-text screening. Any disagreements were resolved through discussion or verified by a third independent reviewer (C Shi) to reach a consensus.

### Data analysis

We extracted the following information from each included trial, including authors, publication years, trial names, NCT numbers, phases, cancer types, HER2-targeted ADCs used, and dosing schedules. As for the primary outcome, the total number of patients who received HER2-targeted ADCs treatment, and the numbers of patients who reported at least one adverse event, including all-grade adverse events, high-grade adverse events, serious adverse events, adverse events that resulted in drug discontinuation, and fatal adverse events, were also extracted for the calculation of incidences. All severity of adverse events was graded on the basis of the CTCAE definitions. High-grade adverse events are defined as grade 3 or higher (grade ≥3). Serious adverse events are defined as any adverse events that: result in death; require hospitalization or prolonged hospitalization; are life-threatening; result in a persistent or significant disability/incapacity. Fatal adverse events are grade-5 adverse events resulting in death. JM Liu and ZW Fu independently did the data extraction. Any discrepancies were resolved by discussion with another senior author (C Shi) to obtain consensus.

The revised Cochrane Risk of Bias tool (RoB version 2.0)^23^ and MINORS criteria^24^ were employed for assessing the risk of bias in each including randomized controlled trials and non-randomized controlled trials, respectively. The incidences were calculated as the number of patients who reported adverse events divided by the total number of patients. The profiles were defined as the incidences of each type of adverse event. Since the incidences of all-grade adverse event and serious adverse event tends towards either 100% or 0%, the variance for that study moves towards zero and as a result, its weight would be overestimated in classic meta-analysis. We hence used the Bayesian hierarchical modelling approach to perform the meta-analysis of all types of adverse events in this study.

Bayesian inference generates posterior distribution to estimate the pooled effect sizes through a prior information and observed sampling distribution.^25^ The response variable (the number of reported adverse events) was assumed to conform to a binomial distribution. With a logit transformation (logit(p) = ln (p/(1-p))) on the incidence probability, we assumed logit transformation of p followed a normal distribution and we could estimate the pooled incidences of adverse events and their corresponding 95% probability intervals (Bayesian credible intervals, CrIs). The CrI represents the 2.5–97.5 percentiles of the posterior distribution of the estimation. Both the mean parameters of normal distributions and the between-study variances (τ) parameters proposed to the noninformative prior distributions. All Bayesian hierarchical models were fitted with the Markov Chain Monte Carlo (MCMC) algorithm and Gibbs sampling to estimate the posterior distribution of interest outcomes.^26^

The heterogeneity among included studies was statistically quantified using the between-study variances (τ) instead of the I^2^ statistic in this Bayesian meta-analysis. The lower value of τ indicates smaller heterogeneity, τ > 1.5 is considered substantial heterogeneity. To explain the between-study variation in the meta-analysis, predefined subgroup analysis were performed considering several study-level moderators, including the cancer type, therapeutic drug and dosing regimen. For sensitivity analyses, we calculated the incidences of adverse events by the removal of the studies with high risks to investigate the robustness of our main analyses. Publication bias was assessed by classic funnel plot and mathematically using Egger's test.^27^ All statistical analyses of extracted data in this study were performed using the R program (version 4.2.0, GUI 1.78, the R Foundation for Statistical Computing) with packages *"R2jags (version 0.7-1)"*, "*coda (version 0.19-4)*", *"bayesmeta (version 3.0)"* and *"forestplot (version 2.0.1)".* The Bayesian meta-analysis methods were detailed in Supplementary file 2.

### Role of the funding source

The funder of the study had no role in study design, data collection, data analysis, data interpretation, or writing of the report. All authors had full access to all the data in the study and had final responsibility for the decision to submit for publication.

## Results

**Fig. 1:**
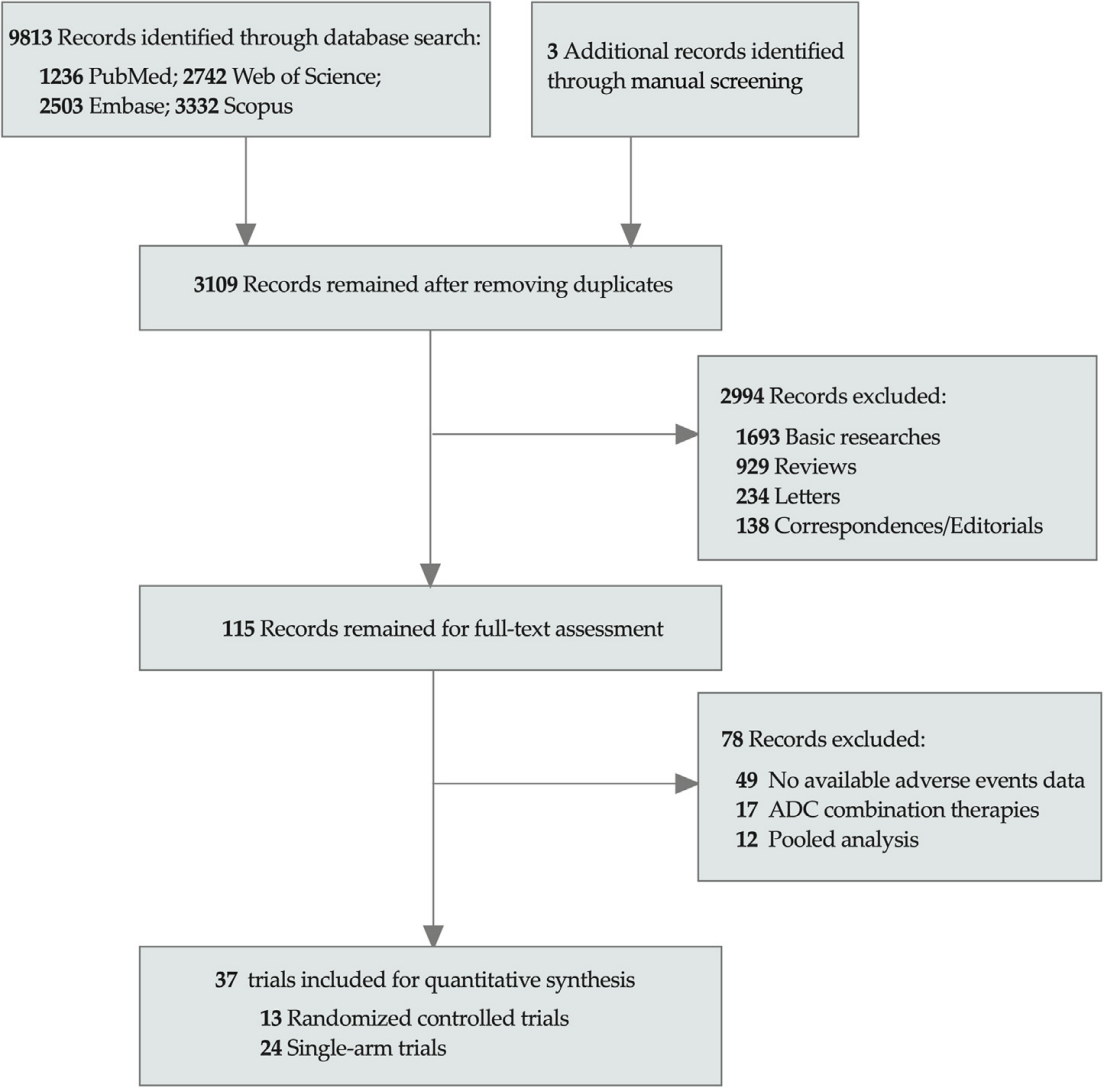
**Flow diagram of the study selection process.** ADC, antibody-drug conjugate.

Our systematic literature search initially identified 9816 relevant records. After the removal of duplicate records, followed by the exclusion of basic researches (n = 1693), review articles (n = 929), editorials (n = 138), and letters (n = 234), 115 records remained. With the evaluation through the full-text articles, we further excluded 78 irrelevant records including articles without adverse events data (n = 49), combination therapies (n = 17), pooled analysis (n = 12), and finally 39 eligible studies (37 trials) involving 7688 patients were included for quantitative analysis (Fig. 1). Of the 37 trials included in the systematic review, 13 were randomized controlled trials (RCTs)^11^^,^^12^^,^^14^^,^^15^^,^^18^^,^^28^, ^29^, ^30^, ^31^, ^32^, ^33^, ^34^, ^35^ and 24 were single-arm clinical trials.^13^^,^^16^^,^^17^^,^^36^, ^37^, ^38^, ^39^, ^40^, ^41^, ^42^, ^43^, ^44^, ^45^, ^46^, ^47^, ^48^, ^49^, ^50^, ^51^, ^52^, ^53^, ^54^, ^55^, ^56^ The HER2-targeted ADCs used in eligible studies included T-DM1 (n = 25) and T-DXd (n = 14). The studies involved the treatment of breast cancer (n = 28), lung cancer (n = 4), gastric cancer (n = 4), colorectal cancer (n = 1), and mixed cancer types (n = 2). Detailed study characteristics were presented in Table 1. The quality assessment showed the included RCTs were almost entirely with a low risk of bias, while only one RCT was evaluated to have a high risk of bias. The MINORS criteria for the non-randomized clinical trials ranged from 13 to 16 for a total score of 16, suggesting that the quality of the included studies was high. The detailed results of risk of bias assessments were available in eTable S1 and eTable S2 in the Supplement.

**Table 1:** Characteristics of included studies.

Author	Year	Study	NCT NO.	Phase	Cancer types	Study types	ADC drug	Dosage	No. of patients	No. of All-grade AEs	No. of Grade>3 AEs	No. of Serious AEs	No. of Discontinuation due to AEs	No. of Death related to AEs
Perez	2019	MARIANNE	NCT01120184	3	Breast cancer	RCT	T-DM1	3.6 mg/kg q3w	361	357	170	164	75	5
Krop	2017	TH3RESA	NCT01419197	3	Breast cancer	RCT	T-DM1	3.6 mg/kg q3w	403	371	161	102	59	9
Cortés	2022	DESTINY-Breast03	NCT03529110	3	Breast cancer	RCT	T-DM1	3.6 mg/kg q3w	261	226	104	16	13	0
Cortés	2020	TRAXHER2	NCT01702558	2	Breast cancer	RCT	T-DM1	3.6 mg/kg q3w	78	69	32	10	12	0
Verma	2012	EMILIA	NCT00829166	3	Breast cancer	RCT	T-DM1	3.6 mg/kg q3w	490	474	233	91	49	4
Minckwitz	2019	KATHERINE	NCT01772472	3	Breast cancer	RCT	T-DM1	3.6 mg/kg q3w	740	731	190	94	133	1
Emens	2020	KATE2	NCT02924883	2	Breast cancer	RCT	T-DM1	3.6 mg/kg q3w	68	62	28	16	11	0
Hurvitz	2013	TDM4450g	NCT00679341	2	Breast cancer	RCT	T-DM1	3.6 mg/kg q3w	69	66	32	14	5	1
Tolaney	2021	ATEMPT	NCT01853748	2	Breast cancer	RCT	T-DM1	3.6 mg/kg q3w	383	383	62	11	67	0
Thungappa	2022	NA	CTRI/2018/07/014881	3	Breast cancer	RCT	T-DM1	3.6 mg/kg q3w	55	41	20	6	3	2
Montemurro	2020	KAMILLA	NCT01702571	3	Breast cancer	Single-Arm	T-DM1	3.6 mg/kg q3w	2002	1862	751	427	237	45
Kashiwaba	2015	JO22997	NA	2	Breast cancer	Single-Arm	T-DM1	3.6 mg/kg q3w	73	70	41	25	14	0
Krop	2011	TDM4374g	NCT00679211	2	Breast cancer	Single-Arm	T-DM1	3.6 mg/kg q3w	110	105	52	29	7	3
Burris III	2010	TDM4258g	NCT00509769	2	Breast cancer	Single-Arm	T-DM1	3.6 mg/kg q3w	112	110	52	30	4	0
Gupta	2013	TDM4688g	NCT00943670	2	Breast cancer	Single-Arm	T-DM1	3.6 mg/kg q3w	51	51	17	4	2	0
WATANABE	2017	JO29317	NA	2	Breast cancer	Single-Arm	T-DM1	3.6 mg/kg q3w	232	228	109	20	5	0
Krop	2015	TDM4874g	NCT01196052	2	Breast cancer	Single-Arm	T-DM1	3.6 mg/kg q3w	148	146	57	15	1	0
Yardley	2015	TDM4884g	NCT01120561	2	Breast cancer	Single-Arm	T-DM1	3.6 mg/kg q3w	215	210	100	20	11	0
Yamamoto	2014	NA	NA	1	Breast cancer	No	T-DM1	Mixed dosage	10	10	3	2	3	0
Beeram	2012	TDM3569g	NCT00932373	1	Breast cancer	No	T-DM1	Mixed dosage	28	25	19	11	4	0
Krop	2010	NA	NA	1	Breast cancer	No	T-DM1	Mixed dosage	24	22	9	1	0	0
Thuss-Patience	2017	GATSBY	NCT01641939	3	Gastric cancer	Yes	T-DM1	2.4 mg/kg weekly	224	218	134	65	31	8
Li^27^	2018	NA	NCT02675829	2	Lung cancer	Single-Arm	T-DM1	3.6 mg/kg q3w	18	18	1	0	0	0
Iwama	2022	NA	NA	2	Lung cancer	Single-Arm	T-DM1	3.6 mg/kg q3w	22	22	7	3	0	1
Hotta	2017	NA	NA	2	Lung cancer	Single-Arm	T-DM1	3.6 mg/kg q3w	15	15	8	3	4	0
Modi	2020	DENSTINY-Breast01	NCT03248492	2	Breast Cancer	No	T-DXd	5.4 mg/kg q3w	184	183	105	42	28	6
Cortes	2022	DENSTINY-Breast03	NCT03529110	3	Breast Cancer	Yes	T-DXd	5.4 mg/kg q3w	257	252	134	49	35	0
Modi	2022	DENSTINY-Breast04	NCT03734029	3	Breast Cancer	Yes	T-DXd	5.4 mg/kg q3w	371	369	195	103	60	7
Siena	2021	DENSTINY-CRC01	NCT03384940	2	Colorectal cancer	No	T-DXd	6.4 mg/kg q3w	78	78	48	26	7	5
Shitara	2020	DENSTINY-Gastric01	NCT03329690	2	Gastric cancer	Yes	T-DXd	6.4 mg/kg q3w	125	125	107	55	19	1
Cutsen	2021	DENSTINY-Gastric02	NCT04014075	2	Gastric cancer	No	T-DXd	6.4 mg/kg q3w	79	79	40	29	12	0
Li	2022	DENSTINY-Lung01	NCT03505710	2	Lung cancer	No	T-DXd	6.4 mg/kg q3w	91	91	63	39	34	2
Bartsch	2022	TUXEDO-1 trial	NCT04752059	2	Breast Cancer	No	T-DXd	5.4 mg/kg q3w	15	15	7	4	3	1
Tsurutani	2020	NA	NCT02564900	1	Mixed Cancer	No	T-DXd	6.4 mg/kg q3w	59	59	37	18	5	2
Modi	2020	NA	NCT02564900	1	Breast Cancer	No	T-DXd	5.4 mg/kg q3w	21	20	11	3	0	0
Modi	2020	NA	NCT02564900	1	Breast Cancer	No	T-DXd	6.4 mg/kg q3w	33	33	23	12	11	3
Tamura	2019	NA	NCT02564900	1	Breast Cancer	No	T-DXd	Mixed dosage	115	115	57	22	13	2
Shitara	2019	NA	NCT02564900	1	Gastric cancer	No	T-DXd	Mixed dosage	44	44	28	11	6	1
Doi	2017	NA	NCT02564900	1	Mixed cancer	No	T-DXd	Mixed dosage	24	24	18	3	2	0

NA, Not applicable; NCT, National Clinical Trial; RCT, Randomized controlled trial; T-DM1, Trastuzumab emtansine; T-DXd, Trastuzumab deruxtecan; AE, adverse event.

**Fig. 2:**
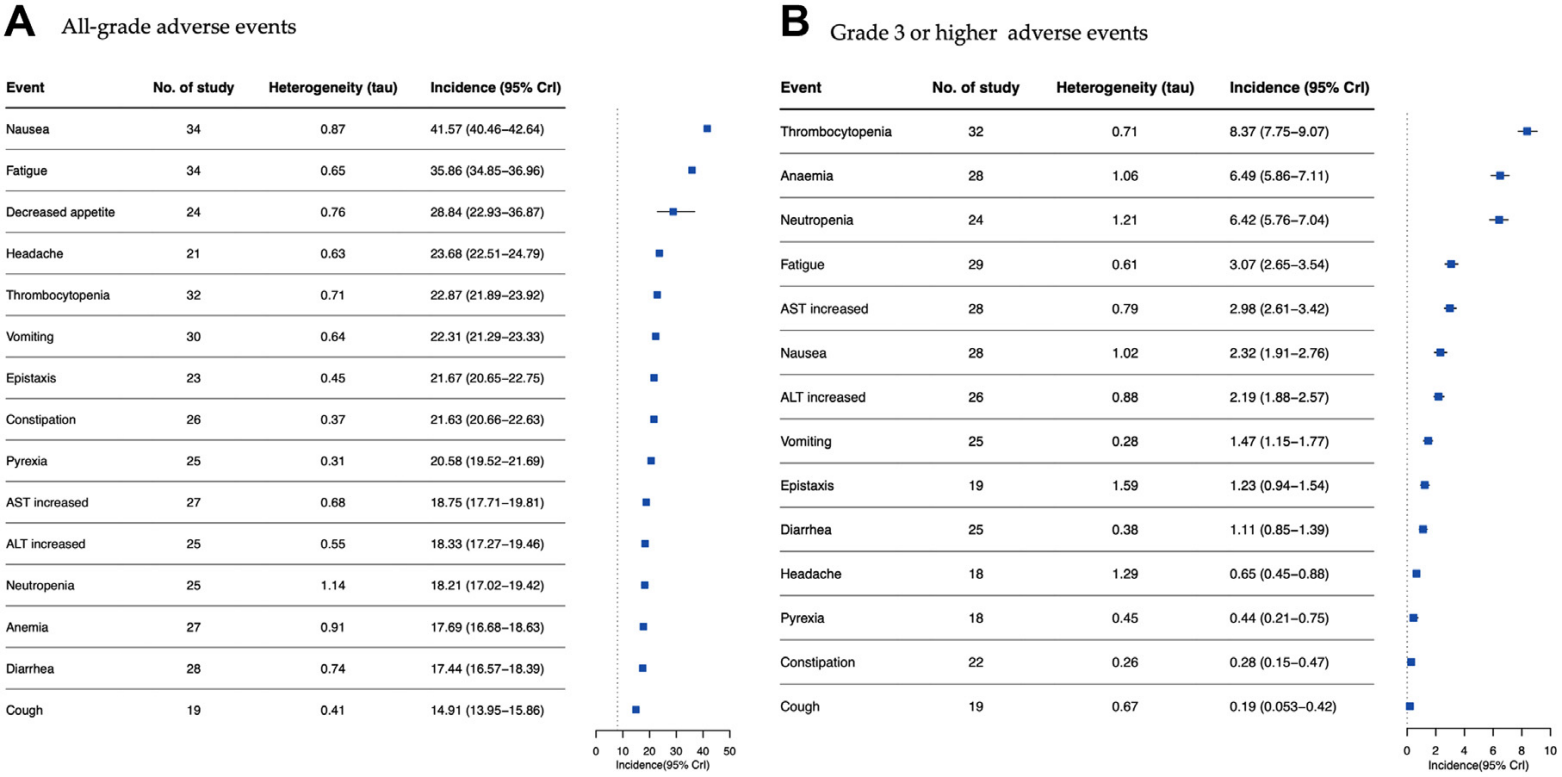
**Incidences of the most common adverse events associated with HER2-targeted antibody-drug conjugates (ADCs).** A, Incidences of the most common all-grade adverse events. B, Incidences of the most common grade 3 or higher adverse events. CrI, Bayesian credible intervals.

On pooling the data using Bayesian hierarchical modelling, the overall mean incidence of all-grade adverse events was 98.29% (95% CrI, 97.33%–99.07%, τ = 1.49). The pooled incidences of high-grade adverse events, serious adverse events, and adverse events that resulted in drug discontinuation were 47.88% (95% CrI, 42.74%–53.17%, τ = 0.37), 19.45% (95% CrI, 15.70%–23.67%, τ = 0.55), and 10.52% (95% CrI, 8.03%–13.21%, τ = 0.56), respectively (eFigs. S1–S4 in the Supplement). In total, over 100 various types of adverse events were reported among 39 included studies, but adverse events that were reported in over half of the included studies were focused in this systematic review to identify the most clinically relevant adverse events. As a result, 15 types of all-grade adverse events and 14 types of high-grade adverse events were presented in Fig. 2. The most common all-grade adverse events were nausea (41.57%; 95% CrI, 40.46%–42.64%, τ = 0.87), fatigue (35.86%; 95% CrI, 34.85%–36.96%, τ = 0.65), decreased appetite (28.84%; 95% CrI, 22.93%–36.87%, τ = 0.76), headache (23.68%; 95% CrI, 22.51%–24.79%, τ = 0.63), and thrombocytopenia (22.87%; 95% CrI, 21.89%–23.92%, τ = 0.71). The most common high-grade adverse events were thrombocytopenia (8.37%; 95% CrI, 7.75%–9.07%, τ = 0.71), anaemia (6.49%; 95% CrI, 5.86%–7.11%, τ = 1.06), neutropenia (6.42%; 95% CrI, 5.76%–7.04%, τ = 1.21), fatigue (3.07%, 95% CrI, 2.65%–3.54%, τ = 0.61), and aspartate aminotransferase (AST) increase (2.98%; 95% CrI, 2.61%–3.42%, τ = 0.79).

To explore the potential severity among the most common adverse events, the incidence ratio of high-grade adverse events to all-grade adverse events was determined as the incidence of high-grade adverse events divided by the respective all-grade adverse events incidence. Adverse event with a higher ratio is considered with a higher possibility to be high-grade when it happens, thereby requiring special attention during HER2-targeted ADC treatment in the clinic. For example, notable among these adverse events were anaemia (incidence ratio, 36.73%), thrombocytopenia (incidence ratio, 36.63%; 95% CrI), neutropenia (incidence ratio, 35.33%), AST increase (incidence ratio, 15.91%), and alanine aminotransferase (ALT) increase (incidence ratio, 12.01%) (eTable S3 in the Supplement). While the most common all-grade adverse events, such as nausea, fatigue, headache, pyrexia, epistaxis, and constipation, had relatively lower risk ratios. These incidence ratios indicate that, although incidences of high-grade hepatotoxicity and haematotoxicity related to HER2-targeted ADC tended to be lower, a higher probability to be high grade whenever these adverse events occurred.

In regards to HER2-targeted ADCs-related death, a total of 109 patients were reported among included studies. The overall incidence of drug-related death was 1.00% (95% CrI, 0.41%–1.78%, τ = 3.84) and the highest incidence (13.09%; 95% CrI, 7.09%–20.56%) was observed in the DENSTINY-Lung 01 trial (NCT03505710).^44^ As summarized in Table 2, pneumonitis (n = 11) and pneumonia (n = 8) were the most common two causes of death representing 17.43% (19/109) of all reported deaths. Other common causes of death associated with HER2-targeted ADCs included interstitial lung disease (n = 7), sepsis (n = 7) and renal failure (n = 3). Other less common causes of death (n = 2) were pulmonary embolism, hepatic failure, respiratory failure, peritonitis, febrile neutropenia, brain oedema, multiple organ dysfunction, and general physical health deterioration. The cause of death is unclear or not reported for the other 31 deaths.

**Table 2:** The detailed cause of death of HER2-targeted antibody-drug conjugates (ADCs) related death in published clinical trials.

Cause of death	Total deaths (n = 109 in 7688 patients)	T-DM1 (n = 79 in 6192 patients)	T-DXd (n = 30 in 1496 patients)
**Respiratory**	**n = 35**	**n = 17**	**n = 18**
Pneumonitis	11	3	8
Pneumonia	8	5	3
Interstitial lung disease	7	2	5
Pulmonary embolism	2	2	0
Respiratory failure	2	1	1
Pneumonia aspiration	1	1	0
Lung infection	1	1	0
Bronchopneumonia	1	1	0
Dyspnea	1	0	1
Atypical pneumonia	1	1	0
**Infectious**	**n=13**	**n=8**	**n=5**
Sepsis	7	4	3
Peritonitis	2	2	0
Septic shock	1	1	0
Neutropenic sepsis	1	1	0
Ischemic colitis	1	0	1
Meningismus	1	0	1
**Hematologic**	**n=11**	**n=8**	**n=3**
Febrile neutropenia	2	1	1
Pulmonary alveolar haemorrhage	1	1	0
Gastric haemorrhage	1	1	0
Upper gastrointestinal haemorrhage	1	1	0
Brain haemorrhage	1	1	0
Subarachnoid haemorrhage	1	1	0
Disseminated intravascular coagulation	1	0	1
Hemorrhagic shock	1	0	1
Decreased platelet count	1	1	0
Acute myeloid leukaemia	1	1	0
**Hepatic**	**n=5**	**n=4**	**n=1**
Hepatic failure	2	2	0
Hepatic encephalopathy	1	1	0
Abnormal hepatic function	1	1	0
Hepatic dysfunction	1	0	1
**Others**	**n=12**	**n=10**	**n=2**
Renal failure	3	3	0
Brain oedema	2	3	0
Multiple organ dysfunction	2	2	0
General physical health deterioration	2	0	2
Metabolic encephalopathy	1	1	0
Acute kidney injury	1	1	0
Acute organ failure	1	0	1
**Unspecific**	**n=33**	**n=33**	**n=0**
Sudden death	1	1	0
Death of unknown cause	1	1	0
Not mentioned	31	31	0

Note: T-DM1, trastuzumab emtansine; T-DXd, trastuzumab deruxtecan.

**Fig. 3:**
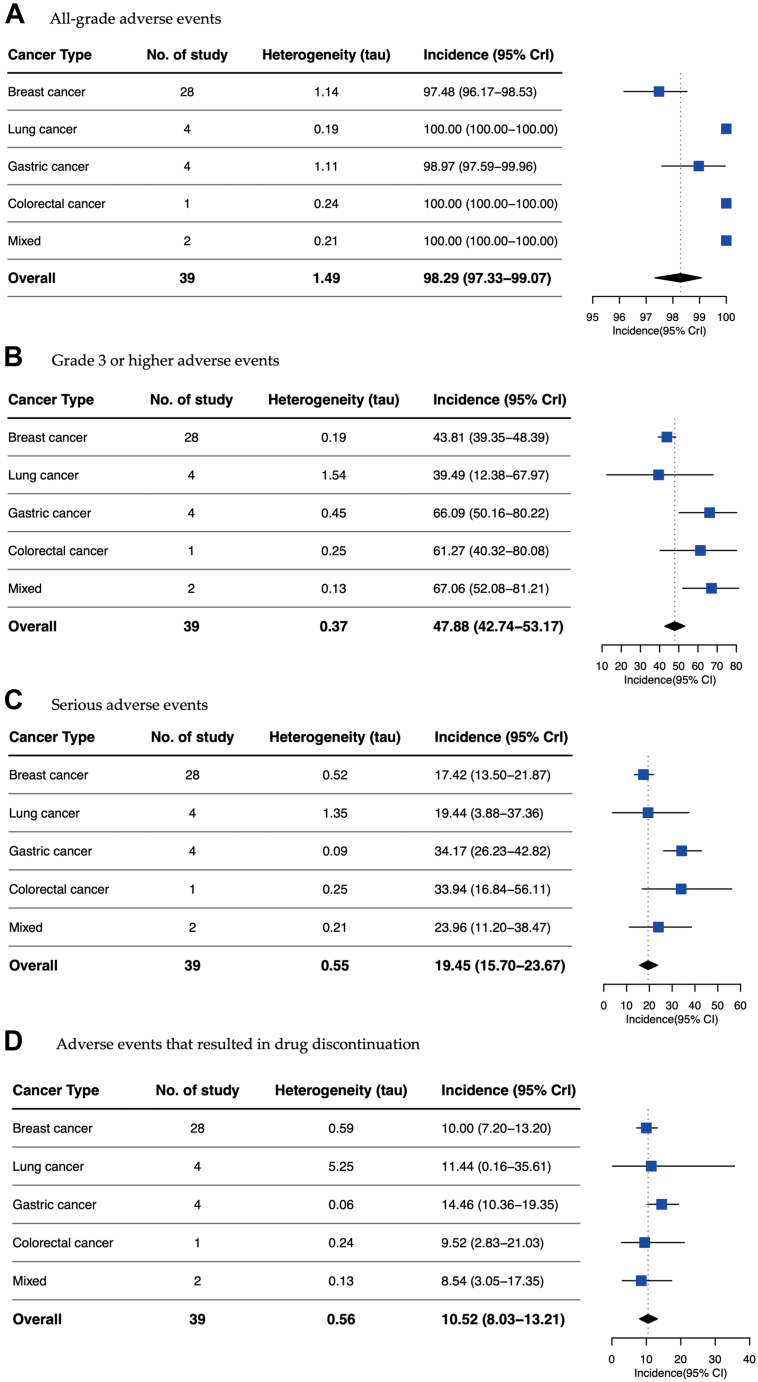
**Mean incidences of adverse events by cancer type.** A, Mean incidences of all grade adverse events by cancer type. B, Mean incidences of grade 3 or higher adverse events by cancer type. C, Mean incidences of serious adverse events by cancer type. D, Mean incidences of adverse events that resulted in drug discontinuation by cancer type. For both panels, values to the left of the line are lower than the mean, to the right, higher. CrI, Bayesian credible intervals.

**Fig. 4:**
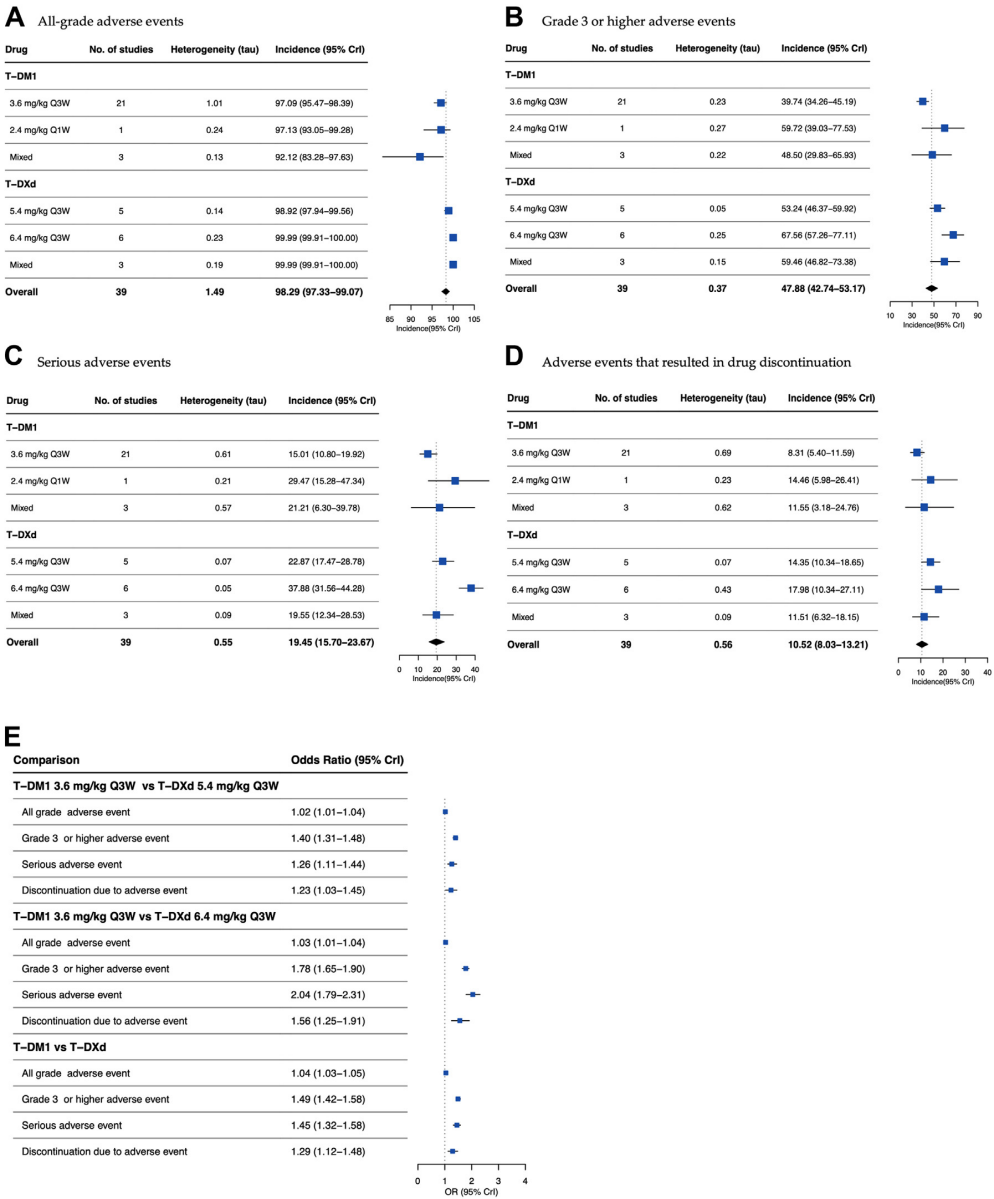
**mean incidences of adverse events by drug type.** A, Mean incidences of all grade adverse events by drug and dose. B, Mean incidences of grade 3 or higher adverse events by drug and dose. C, Mean incidences of serious adverse events by drug and dose. D, Mean incidences of adverse events that resulted in drug discontinuation by drug and dose. E, Comparisons of mean incidences of adverse events between different drugs and dose. CrI, Bayesian credible intervals.

A subgroup analysis by cancer type, treated drug, and dosing regimen was conducted. We found that the mean incidences of all-grade, high-grade adverse events serious adverse events, and drug discontinuation were similar across different cancer types, as well as different dosing regimens of the same drug (Figs. 3 and 4). When comparing the differences between different HER2-targeted ADCs, T-DM1 (3.6 mg/kg every 3 weeks [Q3W] dose) demonstrated lower mean incidences of adverse events compared with two doses of T-DXd (5.4 mg/kg [Q3W] and 6.4 mg/kg [Q3W]). As shown in Fig. 4E, the highest odds ratio (OR) was observed in the comparison group of T-DM1 (3.6 mg/kg [Q3W]) and T-DXd (5.4 mg/kg [Q3W]) for the mean incidence of serious adverse event (2.04; 95% CrI, 1.79–2.31). The ORs in the same comparison group of the mean incidences of high-grade adverse event and drug discontinuation due to adverse event were 1.78 (95% CrI, 1.65–1.90) and 1.56 (95% CrI, 1.25–1.91), respectively. In addition, the overall mean incidences of adverse events of T-DXd was also higher than T-DM1, the ORs of high-grade adverse event and serious adverse event were 1.49 (95% CrI, 1.42–1.58) and 1.45 (95% CrI, 1.32–1.58), respectively.

We evaluated publication bias by generating funnel plots incorporating estimated heterogeneity in the different studies. Analysis of all included studies for mean incidences of all types of adverse events showed a symmetrical distribution of the studies, providing no evidence of publication bias (eFigs. S6–S9 in the Supplement). The results were further confirmed by Egger's test (p > 0.1). As for sensitivity analyses, one RCT with a high risk of bias was removed, and the estimated pooled incidences of all-grade adverse events, high-grade adverse events, serious adverse events, and adverse events that resulted in drug discontinuation were 98.51% (95% CrI, 97.54%–99.30%, τ = 1.99), 48.19% (95% CrI, 42.75%–53.88%, τ = 0.42), 19.68% (95% CrI, 15.46%–24.30%, τ = 0.62), and 10.55% (95% CrI, 7.80%–13.58%, τ = 0.67), respectively (eTable S4 in the Supplement). These pooled overall incidences were similar to those of all-included analysis except for wider credible intervals, suggesting our estimated incidences were robust and conservative.

## Discussion

In this work, we performed a systematic review of HER2-targeted ADCs associated with adverse events using a Bayesian hierarchical modelling approach. We collected all adverse event data including the sparse binomial data from all published clinical trials for the meta-analysis. This meta-analysis used the number of each treatment-related adverse event to generate exact statistical inferences, which were close to the results from individual-level data.^57^ It is always challenging to get and combine original patient data at the individual level, but a feasible approach for estimating study moderator effects was provided in this meta-analysis without loss of relative efficiency. Toxicity profiles are important factors to consider when obtaining informed consent from patients and a clear understanding of HER2-ADCs associated adverse events is necessary to assess the benefit-risk ratio and enable their proper management. Therefore, we focused on the summarization of the toxicity profiles of approved HER2-ADCs to provide reference for clinicians to prescribe HER2-ADCs. Compared with the previous published reports,^19^, ^20^, ^21^ T-DXd was included in our analysis and more prospective clinical trials were included for the pooled analysis of incidences.

Our work suggests that almost all (98.29%) patients receiving HER2-targeted ADCs experienced at least one adverse event, of which 47.88% of patients had at least one high-grade adverse event and 19.45% of patients suffered from serious adverse events. The incidence of adverse events that resulted in the discontinuation of HER2-targeted ADCs treatment occurred in 10.52% of patients. These mean incidences of adverse events information would be crucial to inform patients when they adopt HER2-targeted ADCs therapy for cancer treatment. Similar to standard chemotherapies, nausea (41.57%) and fatigue (35.86%) accounted for the two most common all-grade adverse events associated with HER2-targeted ADCs, however, the grades were commonly low (grade 1–2). The most frequently occurring high-grade adverse event was thrombocytopenia (8.37%), anaemia (6.49%), and neutropenia (6.42%) that needs a special concern during HER2-targeted ADCs treatment.

The incidence ratio of high-grade adverse events to all-grade adverse events revealed thrombocytopenia, neutropenia, anaemia, ALT increase, and AST increase tend to be severe (a higher probability to be high grade when these adverse events occurred). Clinicians should pay close attention to haematological toxicity and hepatoxicity and monitor possible clinical symptoms of these adverse events during the use of HER2-targeted ADCs, especially in patients with underlying known hepatic disease or hematopoietic dysfunction. Our meta-analysis also demonstrated that approximately 1.0% (95% CrI, 0.41%–1.78%, τ = 3.84) of patients treated with HER2-targeted ADCs have a risk of a drug-related death. The differences in the cause of death between two drugs have possibly contributed to the high between-study variance (τ). We hence separately summarized the cause of death for T-DM1 and T-DXd. It is also worth noting that the respiratory problems, particularly pneumonitis and interstitial lung disease, are more serious in patients receiving T-DXd than T-DM1, which need keep close monitoring of respiratory symptoms in patients receiving T-DXd.

Through the subgroup analysis, the results indicated no difference in the mean incidences of adverse events among different cancer types, as well as different dosing regimens. It may probably be because the doses of HER2-ADCs used currently in clinic are limited and the undesired toxicity of HER2-ADCs is caused by the independent uptake of the ADC in healthy cells.^58^ Nevertheless, T-DXd appeared to have higher mean incidences of adverse events compared with T-DM1, especially for the higher dose of T-DXd (6.4 mg/kg Q3W). Compared to the molecular design of T-DM1, T-DXd achieved breakthroughs and innovations in the linker-payload system. Utilizing a protease-cleavable linker, a more potent payload, and a higher drug antibody ratio (DAR), T-DXd is able to elicit a potent bystander effect and increase the anticancer efficacy.^59^, ^60^, ^61^ However, the improvement in the molecular design of T-DXd is like a "double-edged sword". As well as enhancing the potency for anticancer treatment, it possibly brings a higher mean incidence of treatment-related adverse events. While we marvel at the excellent efficacy data of T-DXd, we also need to pay attention to its potential safety issue, which is also one of the key directions for the development of the next generation of HER2-targeted ADC drugs.

To the best of our knowledge, this is the first and the most comprehensive meta-analysis of treatment-related adverse events for HER2-targeted ADCs in patients with cancer. Our findings should have important implications for populations across multiple fields. With the increasing use of HER2-targeted ADCs worldwide, the results from this meta-analysis are important from the standpoint of patient counselling. Clinicians could share the information of the incidences and toxicity profiles of HER2-ADCs with patients before they begin treatment with a HER2-ADC. In addition to severe adverse events such as hepatotoxicity, hematologic toxicity, and pneumonitis, our study suggests these problems that will require management by a multidisciplinary team. Multidisciplinary clinical teams may better meet the long-term needs of patients for the early recognition and proper management of serious adverse events. As for researchers who engaged in the development of HER2-ADC drugs, the information of treatment-related adverse events would be also important for the optimization of next generation of HER2-ADCs.

The principle advantage of our work is the use of the Bayesian hierarchical modelling approach. Compared with the classical frequency-based meta-analysis, the Bayesian hierarchical modelling approach has the following advantages: (i) The modelling is more flexible and highly implementable; (ii) The results are more reliable because the Bayesian approach allows us to account for uncertainty from the varying quality of data and borrow strength from non-missing data, and MCMC sampling allows for inference in a high-dimensional, constrained parameter space, while providing posterior estimation that allows straightforward inference on the wide variety of functionals of interest. (iii) The zero data was allowed in the binomial likelihood to avoid continuity correction for sparse binomial data.

However, several limitations also existed in this meta-analysis. Firstly, the grade information of adverse events was extracted from different multicentre trials using different versions of CTCAE, resulting in a bias of reporting some adverse events grading in the publication. For example, EMILIA and TDM4450g used CTCAE version 3.0, DESTINY-Breast03 used CTCAE version 5.0, while MARIANNE, TH3RESA, and TRAXHER2 adopted CTCAE version 4.0. In addition, most of the included trials were open-label trials. Although the quality assessment showed low risks of bias, when participants and investigators are both aware of the drug or treatment allocation, it might cause extra bias in the reporting of the incidence of adverse events. Finally, whether some adverse events are more likely to be found in specific cancer types has not been investigated in this meta-analysis, and it might be one of the focuses of follow-up studies in the future.

In conclusion, using a Bayesian hierarchical modelling approach, our systematic review and meta-analysis firstly revealed the overall incidences of treatment-related adverse events of HER2-targeted ADCs in patients with cancer in clinical trials. The incidences of adverse events between two HER2-targeted ADCs were similar in different cancer types, but T-DXd appeared to have higher mean incidences of adverse events compared with T-DM1. The comprehensive summary of treatment-related adverse events associated with HER2-targeted ADCs is critical for clinicians caring for patients with cancer receiving HER2-targeted ADCs therapy.

## Contributors

ZF and CS contributed to the conceptualization of the study, development of methodology, and data analysis. CS provided funding and support for this study. ZF, JL, SL, and CS performed the database searches and reference review. ZF, SL, and JL contributed to the assessment of study quality and data interpretation. ZF and CS wrote the original draft of the manuscript. CS and YZ helped to review and write the final report. ZF and CS accessed and verified the underlying data.

## Data sharing statement

All the original data of this study are available with publication from the corresponding authors: 29136909@qq.com (C. Shi); whxhzy@163.com (Y. Zhang) for the similar toxicity profile analyses associated with HER2-targeted ADCs.

## Declaration of interests

All authors declare no competing interests.

## Appendix A Supplementary data

Supplementary files, Figures, and Tables

Translated Abstract in Chinese
