# Supplementary material for: Treatment-related adverse events associated with HER2-Targeted antibody-drug conjugates in clinical trials: a systematic review and meta-analysis
Source: eClinicalMedicine. 2022 Dec 27;55:101795. doi: 10.1016/j.eclinm.2022.101795 (PMC9874347; doi:10.1016/j.eclinm.2022.101795)
Supplement: Supplementary files, Figures, and Tables [file mmc1.pdf]

Supplement

Treatment-Related Adverse Events Associated with HER2-targeted Antibody-Drug Conjugates in Clinical Trials

A Systematic Review and Meta-analysis

Zhiwen Fu, PhD[1,2]; Jinmei Liu, MPhil [1,2]; Shijun Li, MPhil [1,2]; Chen Shi, PhD[1,2]; Yu Zhang, PhD[1,2]

[1]Department of Pharmacy, Union Hospital, Tongji Medical College, Huazhong University of Science and Technology;

[2] Hubei Province Clinical Research Centre for Precision Medicine for Critical Illness

Corresponding authors: Chen Shi and Yu Zhang; Department of Pharmacy, Union Hospital, Tongji Medical College, Huazhong University of Science and Technology, 1277 Jiefang Avenue, Jianghan District, Wuhan, Hubei Province, China, 430000; E-mail: 29136909@qq.com (C. Shi); whxhzy@163.com (Y. Zhang); Tel: +86-27-85726192 (C. Shi); +86-27- 85726399 (Y. Zhang)

Contents

Supplementary Files *3*

Supplementary file 1: Search strategy 3

Supplementary file 2: Bayesian hierarchical model and the code from R software 5

3.1 The Bayesian hierarchical model 5

3.2 Parameter Estimation 6

3.3 Code of "R2jags" package from R software 6

Supplementary Tables *8*

eTable 1. The RoB-2 tool[4] for assessing the quality of included Randomized Controlled Trials (n=13) 8

eTable 2. The MINORS tool [5] for assessing the quality of included Non- Randomized Controlled Trials (n = 24) 9

eTable 3. Incidence Ratio of High-grade to All-grade Adverse Events 10

eTable 4. Estimated pooled incidence of adverse events associated with HER2-targeted ADCs for sensitivity analysis 11

Supplementary Figures *12*

eFigure 1. Overall incidences of all-grade adverse events associated with HER2-targeted ADCs (heterogeneity $\tau = 1.89$) 12

eFigure 2. Overall incidences of grade 3 or higher adverse events associated with HER2-targeted ADCs (heterogeneity $\tau = 0.42$) 13

eFigure 3. Overall incidences of serious adverse events associated with HER2-targeted ADCs (heterogeneity $\tau = 0.61$) 14

eFigure 4. Overall incidences of adverse events that resulted in drug discontinuation associated with HER2-targeted ADCs (heterogeneity $\tau = 0.67$) 15

eFigure 5. Overall incidences of fatal adverse events associated with HER2-targeted ADCs (heterogeneity $\tau = 3.84$) 16

eFigure 6. Funnel plot of the overall incidence of all-grade adverse events (Egger's test: p= 0.136). 17

eFigure 7. Funnel plot of the overall incidence of grade 3 or higher adverse events (Egger's test: p= 0.259). 17

eFigure 8. Funnel plot of the overall incidence of serious adverse events (Egger's test: p= 0.204). 18

eFigure 9. Funnel plot of the overall incidence of adverse events that resulted in drug discontinuation (Egger's test: p= 0.153). 18

References *19*

Supplementary Files

Supplementary file 1: Search strategy

1. PubMed

Access Date: 14 August 2022

Search strategy: Ado Trastuzumab Emtansine[Title/Abstract] OR Trastuzumab Emtansine[Title/Abstract] OR Kadcyla[Title/Abstract] OR Trastuzumab DM1 Conjugate[Title/Abstract] OR Trastuzumab DM1[Title/Abstract] OR T-DM1[Title/Abstract] OR trastuzumab deruxtecan[Title/Abstract] OR DS-8201[Title/Abstract] OR DS-8201a[Title/Abstract] OR T-Dxd[Title/Abstract]

Search Results: 1236

2. Web of Science

Access Date: 14 August 2022

Search strategy: TS= (Ado Trastuzumab Emtansine OR Trastuzumab Emtansine OR Kadcyla OR Trastuzumab DM1 OR T-DM1 OR Trastuzumab DM1 Conjugate OR trastuzumab deruxtecan OR DS-8201 OR DS-8201a OR T-Dxd)

Search Results: 2742

3. Embase

Access Date: 14 August 2022

Search strategy: 'Ado Trastuzumab Emtansine':ab,ti OR 'Trastuzumab Emtansine':ab,ti OR 'Kadcyla':ab,ti OR 'Trastuzumab DM1 Conjugate':ab,ti OR 'Trastuzumab DM1':ab,ti OR 'T-DM1':ab,ti OR 'trastuzumab deruxtecan':ab,ti OR 'DS-8201':ab,ti OR 'DS-8201a':ab,ti OR 'T-Dxd':ab,ti

Search Results: 2503

4. Scopus

Access Date: 14 August 2022

Search strategy: TITLE-ABS-KEY("Ado Trastuzumab Emtansine" OR "Trastuzumab Emtansine" OR "Kadcyla" OR "T-DM1" OR "Trastuzumab DM1" OR "Trastuzumab DM1 Conjugate" OR "trastuzumab deruxtecan" OR "DS-8201" OR "DS-8201a" OR "T-Dxd")

Search Results: 3332

Supplementary file 2: Bayesian hierarchical model and the code from R software

2.1 The Bayesian hierarchical model

Meta-analysis usually combines aggregated or individual results from several studies to create a pooled, more precise estimate of an effect. Due to the hierarchical structure (intra-study and between-study) of meta-analysis data, the Bayesian hierarchical approach is often used in meta-analysis [1,2]. Meanwhile, the Bayesian approach allows us to account for uncertainty from the varying quality of data and borrow strength from non-missing data, and MCMC sampling allows for inference in a high-dimensional, constrained parameter space, while providing posterior estimation that allow straightforward inference on the wide variety of functionals of interest. In our meta-analysis, the outcomes of interest were the incidences of adverse events associated with HER2-targeted antibody-drug conjugates in clinical trials. We use the Bayesian Hierarchical models to estimate the pooled incidences of all-grade adverse events, high-grade (grade 3 or higher) adverse events, serious adverse events, and adverse events that resulted in drug discontinuation.

For the ith study which reported the dichotomous outcomes, the number of patients with reported any adverse events in the ith study followed the binomial distribution:

$$r_i \sim \text{binomial}\ (n_i\ ,\ p_i) \qquad (1)$$

Where n_i was the total number of investigated population and p_i was the incidence of adverse event for the ith study.

The logit transformation of p_i followed a normal distribution among studies:

$$\theta_i = \text{logit}(\ p_i\ ) \sim \text{normal}\ (\mu,\ \sigma^2\ ) \qquad (2)$$

Where μ was the mean of logit(p_i) and σ^2 was the between-study variance.

Then we could estimate the pooled incidence of adverse event and the corresponding 95% credible interval (CrI) through retransform the

$$Incidence = \exp(\mu)/(1 + exp(\mu)) \qquad (3)$$

2.2 Parameter Estimation

All Bayesian Hierarchical models were fitted with the Markov chain Monte Carlo (MCMC) algorithm and Gibbs sampling to estimate the posterior distribution of the outcomes [3]. Non-informative prior was specified for all the parameters. The credible intervals (or CrI) represent the 2.5-97.5 percentiles of the posterior distribution of the estimation. Inferences were based on 5000 iterations, and the first 2500 of which were used as burn-in. All the Bayesian Hierarchical analyses were performed using the "R2jags" package of R software (version 4.0.3). In the BUGS model, normal distribution was written as mean and precision $\tau^2 = 1/\sigma^2$, which was not the variance σ^2 .

2.3 Code of "R2jags" package from R software

```
library(R2jags)
library(mcmcplots)
set.seed(12345)
data = read.csv
# write bugs model in R as a function
bayesmodel.1<-function(){
 for (i in 1:N){ # N, the number of studies
   r[i] ~ dbinom (p[i], n[i]) # data model
   logit(p[i])<-y[i] # the logit transformation for p
 }
```

```
 for (i in 1:N){
   y[i] ~ dnorm (mu, tau) # hierarchical model for y
 }
 tau <- pow(sigma, -2) # tau = 1/sigma^2
 mu ~ dnorm (0.0, 1.0E-6) # non-informative prior on mu
 sigma ~ dunif (0, 1000) # non-informative prior on sigma
 incidence<-exp(mu)/(1+exp(mu)) # the pooled incidence
 sigma2<-sigma*sigma
}
jags.params.1 <-c("mu","sigma2","p","incidence")
jags.1 <- jags(data=list(N=length(data$Studyid),r=data$count1,n=data$n),
          inits=NULL, jags.params.1, n.iter=5000, model.file=bayesmodel.1)
jags.1
```

Supplementary Tables

eTable 1. The RoB-2 tool[4] for assessing the quality of included Randomized Controlled Trials (n=13)

Study	Randomization	Allocation concealment	Blinding of participants and staff	Blinding of outcome assessors	Incomplete outcome data	Selective reporting	Other bias	Overall bias
Cortés 2022	Low	Low	Low	Low	Low	Low	Low	Low
Cortés 2020	Low	Low	Low	Low	Low	Low	Low	Low
Emens 2020	Low	Low	Low	Low	Low	Low	Low	Low
Hurvitz 2013	Low	Low	Low	Low	Low	Low	Low	Low
Krop 2017	Low	Low	Low	Low	Low	Low	Low	Low
Minckwitz 2019	Low	Low	Low	Low	Low	Low	Low	Low
Modi 2022	Low	Low	Low	Low	Low	Low	Low	Low
Perez 2019	Low	Low	Low	Low	Low	Low	Low	Low
Shitara 2020	Low	Low	Low	Low	Low	Low	Low	Low
Thungappa 2022	Low	Some concerns	Some concerns	Some concerns	Low	Low	Some concerns	High
Thuss-Patience 2017	Low	Low	Low	Low	Low	Low	Low	Low
Tolaney 2021	Low	Low	Low	Low	Low	Low	Low	Low
Verma 2012	Low	Low	Low	Low	Low	Low	Low	Low

Rob-2: Version 2 of the Cochrane risk-of-bias tool for randomized trials

eTable 2. The MINORS tool [5] for assessing the quality of included Non- Randomized Controlled Trials (n = 24)

Study	A clearly stated aim	Inclusion of consecutive patients	Prospective collection of data	Endpoints appropriate to the aim of the study	Unbiased assessment of the study endpoint	Follow-up period appropriate to the aim of the study	Loss to follow up less than 5%	Prospective calculation of the study size	Total Quality Score
Li 2022	2	2	2	2	2	2	2	2	16
Modi 2020	2	2	2	2	2	2	2	2	16
Bartsch 2022	2	2	2	2	2	2	2	2	16
Beeram 2012	2	2	1	2	2	1	2	1	13
Burris III 2010	2	2	2	2	2	2	2	2	16
Cutsen 2021	2	2	2	2	2	1	2	2	15
Doi 2017	2	2	2	2	2	1	2	1	14
Gupta 2013	2	2	2	2	2	1	2	1	14
Hotta 2017	2	2	2	2	2	2	2	2	16
Iwama 2022	2	2	2	2	2	2	2	2	16
Kashiwaba 2015	2	2	2	2	2	2	2	2	16
Krop 2011	2	2	2	2	2	1	2	1	14
Krop 2015	2	2	2	2	2	2	2	2	16
Krop 2010	2	2	2	2	2	2	2	2	16
Li 2018	2	2	2	2	2	2	2	2	16
Modi 2020	2	2	2	2	2	1	2	1	14
Montemurro 2020	2	2	2	2	2	2	2	2	16
Shitara 2019	2	2	2	2	2	2	2	2	16
Siena 2021	2	2	2	2	2	2	2	2	16
Tamura 2019	2	2	2	2	2	2	2	2	16
Tsurutani 2020	2	2	2	2	2	2	2	2	16
Yamamoto 2014	2	2	2	2	2	1	2	1	14
Watanabe 2017	2	2	1	2	2	1	2	1	13
Yardley 2015	2	2	2	2	2	2	2	2	16

MINORs: Methodological index for non-randomized studies. The items are scored 0 (not reported), 1 (reported but inadequate) or 2 (reported and adequate).

eTable 3. Incidence Ratio of High-grade to All-grade Adverse Events

AE type	Incidence of all-grade AE (95% CrI)	Incidence of high-grade AE (95% CrI)	Incidence Ratio(95% CrI)
Anemia	17.69 (16.68-18.63)	6.49 (5.86-7.11)	36.73 (32.74-41.49)
Thrombocytopenia	22.87 (21.89-23.92)	8.37 (7.75-9.07)	36.63 (33.39-40.16)
Neutropenia	18.21 (17.02-19.42)	6.42 (5.76-7.04)	35.33 (31.11-39.51)
AST increased	18.75 (17.71-19.81)	2.98 (2.61-3.42)	15.91 (13.79-18.32)
ALT increased	18.33 (17.27-19.46)	2.19 (1.88-2.57)	12.01 (10.13-14.21)
Fatigue	35.86 (34.85-36.96)	3.07 (2.65-3.54)	8.56 (7.35-9.79)
Vomiting	22.31 (21.29-23.33)	2.19 (1.88-2.57)	6.58 (5.14-7.95)
Diarrhea	17.44 (16.57-18.39)	1.11 (0.85-1.39)	6.32 (4.78-8.09)
Epistaxis	21.67 (20.65-22.75)	1.23 (0.94-1.54)	5.67 (4.27-7.19)
Nausea	41.57 (40.46-42.64)	2.32 (1.91-2.76)	5.58 (4.61-6.68)
Headache	23.68 (22.51-24.79)	0.65 (0.45-0.88)	2.73 (1.89-3.76)
Pyrexia	20.58 (19.52-21.69)	0.44 (0.21-0.75)	2.14 (1.01-3.65)
Constipation	21.63 (20.66-22.63)	0.28 (0.15-0.47)	1.31 (0.69-2.19)
Cough	14.91 (13.95-15.86)	0.28 (0.15-0.47)	1.31 (0.36-2.81)
Decreased appetite	28.84 (22.93-36.87)	0.17 (0.11-0.23)	0.59 (0.22-0.98)

AE: adverse events, AST: Aspartate transaminase, ALT: alanine aminotransferase

eTable 4. Estimated pooled incidence of adverse events associated with HER2-targeted ADCs for sensitivity analysis

Criteria	Estimated pooled incidence (mean, [95% CrI])			
	All-grade adverse events	Grade 3 or higher adverse events	Serious adverse events	Adverse events that resulted in drug discontinuation
All-included meta-analysis	98.29% (97.33%-99.07%, τ=1.49)	47.88% (42.74%-53.17%, τ=0.37)	19.45% (15.70%-23.67%, τ=0.55)	10.52% (8.03%-13.21%,τ=0.56)
Removing study with high risk of bias	98.51% (97.54%–99.30%, τ=1.99)	48.19% (42.75%–53.88%, τ=0.42)	19.68% (15.46%-24.30%, τ=0.62)	10.55% (7.80%-13.58%, τ=0.67)

CrI: credible interval; τ: between-study variance which used to indicate heterogeneity

Supplementary Figures

eFigure 1. Overall incidences of all-grade adverse events associated with HER2-targeted ADCs (heterogeneity τ = 1.89)

Study	Incidence (95% CrI)
Perez 2019	98.79 (97.52−99.60)
Krop 2017	92.36 (89.59−94.70)
Cortés 2022	87.25 (83.04−90.97)
Cortés 2020	90.22 (83.04−95.52)
Verma 2012	96.83 (95.15−98.14)
Minckwitz 2019	98.75 (97.88−99.40)
Emens 2020	92.83 (86.04−97.18)
Hurvitz 2013	96.33 (91.36−99.05)
Tolaney 2021	99.65 (98.96−99.96)
Thungappa 2022	94.27 (87.65−98.29)
Montemurro 2020	93.06 (91.88−94.13)
Kashiwaba 2015	96.48 (91.63−99.09)
Krop 2011	96.01 (91.90−98.65)
Burris III 2010	98.16 (95.48−99.61)
Gupta 2013	98.88 (96.10−99.91)
WATANABE 2017	98.24 (96.35−99.42)
Krop 2015	98.49 (96.34−99.68)
Yardley 2015	97.72 (95.58−99.17)
Yamamoto 2014	97.92 (91.68−99.89)
Beeram 2012	92.75 (82.05−98.47)
Krop 2010	94.62 (85.04−99.12)
Thuss−Patience 2017	97.42 (95.15−98.95)
Li 2018	98.27 (93.60−99.90)
Iwama 2022	98.41 (93.81−99.90)
Hotta 2017	98.20 (93.11−99.90)
Modi 2020	99.11 (97.68−99.86)
Cortes 2022	98.09 (96.24−99.28)
Modi 2022	99.26 (98.27−99.82)
Siena 2021	99.10 (96.96−99.92)
Shitara 2020	99.30 (97.79−99.93)
Cutsen 2021	99.09 (96.95−99.93)
Li 2022	99.16 (97.30−99.93)
Bartsch 2022	98.18 (92.83−99.89)
Tsurutani 2020	98.95 (96.44−99.92)
Modi 2020	96.67 (89.66−99.62)
Modi 2020	98.66 (95.05−99.91)
Tamura 2019	99.25 (97.52−99.93)
Shitara 2019	98.82 (95.76−99.92)
Doi 2017	98.47 (94.31−99.91)
**Overall**	**98.29 (97.33−99.07)**

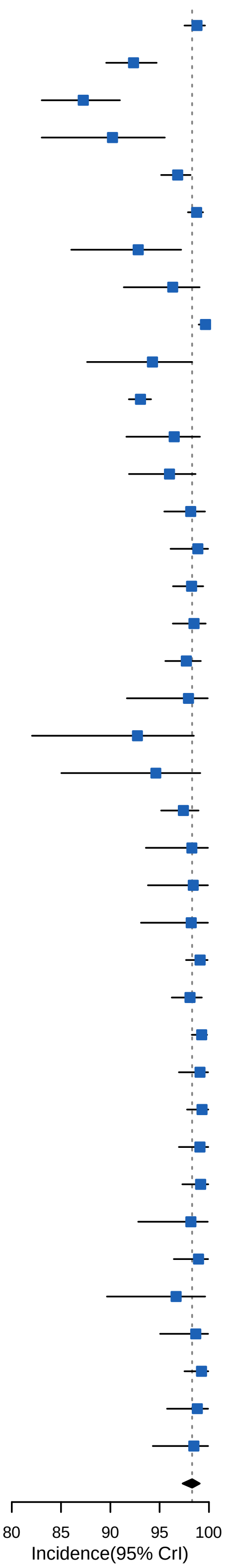

eFigure 2. Overall incidences of grade 3 or higher adverse events associated with HER2-targeted ADCs (heterogeneity τ = 0.42)

Study	Incidence (95% CrI)
Perez 2019	47.08 (41.91−52.07)
Krop 2017	40.17 (35.56−44.89)
Cortés 2022	40.21 (34.45−45.99)
Cortés 2020	41.92 (31.86−52.19)
Verma 2012	47.55 (43.18−52.10)
Minckwitz 2019	26.05 (23.00−29.23)
Emens 2020	42.16 (31.79−53.07)
Hurvitz 2013	46.59 (36.06−57.24)
Tolaney 2021	17.29 (13.77−21.20)
Thungappa 2022	38.41 (27.22−50.60)
Montemurro 2020	37.59 (35.47−39.75)
Kashiwaba 2015	54.93 (44.27−65.30)
Krop 2011	47.37 (38.51−56.19)
Burris III 2010	46.58 (37.90−55.39)
Gupta 2013	36.17 (24.83−48.36)
WATANABE 2017	47.01 (40.84−53.13)
Krop 2015	39.22 (31.86−46.70)
Yardley 2015	46.61 (40.18−53.10)
Yamamoto 2014	39.69 (20.99−60.20)
Beeram 2012	61.91 (46.44−76.49)
Krop 2010	41.28 (25.67−57.29)
Thuss−Patience 2017	59.25 (52.85−65.57)
Li 2018	23.54 (10.32−40.07)
Iwama 2022	37.69 (22.42−54.21)
Hotta 2017	50.96 (32.26−69.69)
Modi 2020	56.45 (49.47−63.27)
Cortes 2022	52.10 (46.17−57.96)
Modi 2022	52.44 (47.44−57.59)
Siena 2021	59.79 (49.61−69.46)
Shitara 2020	81.88 (74.79−87.89)
Cutsen 2021	50.33 (40.15−60.62)
Li 2022	66.75 (57.29−75.36)
Bartsch 2022	47.21 (28.91−65.83)
Tsurutani 2020	60.25 (48.40−71.38)
Modi 2020	51.03 (34.74−67.77)
Modi 2020	64.06 (49.95−77.02)
Tamura 2019	49.43 (40.91−58.13)
Shitara 2019	60.49 (47.33−73.05)
Doi 2017	65.95 (49.39−80.69)
**Overall**	**47.88 (42.74−53.17)**

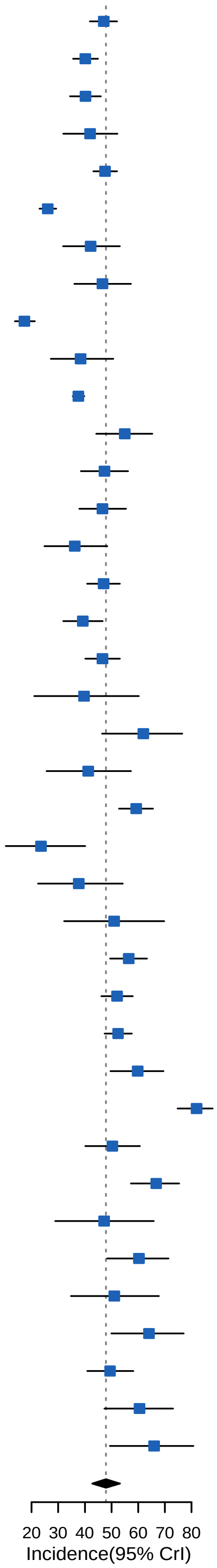

eFigure 3. Overall incidences of serious adverse events associated with HER2-targeted ADCs (heterogeneity τ = 0.61)

Study	Incidence (95% CrI)
Perez 2019	44.73 (39.75−49.96)
Krop 2017	25.15 (21.16−29.41)
Cortés 2022	7.01 (4.32−10.21)
Cortés 2020	13.86 (7.84−21.57)
Verma 2012	18.58 (15.32−21.93)
Minckwitz 2019	12.80 (10.56−15.30)
Emens 2020	22.92 (14.38−32.79)
Hurvitz 2013	20.25 (12.33−29.61)
Tolaney 2021	3.81 (2.13−5.85)
Thungappa 2022	12.87 (6.18−21.53)
Montemurro 2020	21.31 (19.58−23.09)
Kashiwaba 2015	32.37 (22.46−42.93)
Krop 2011	25.73 (18.22−33.77)
Burris III 2010	26.20 (18.75−34.49)
Gupta 2013	10.65 (4.61−19.00)
WATANABE 2017	9.36 (6.05−13.39)
Krop 2015	11.01 (6.71−16.16)
Yardley 2015	10.03 (6.58−14.13)
Yamamoto 2014	20.31 (6.83−39.98)
Beeram 2012	34.03 (20.26−49.85)
Krop 2010	10.62 (3.51−21.87)
Thuss−Patience 2017	28.63 (23.24−34.60)
Li 2018	9.76 (2.70−21.78)
Iwama 2022	16.30 (6.33−30.12)
Hotta 2017	20.31 (8.05−38.27)
Modi 2020	22.68 (17.16−28.86)
Cortes 2022	19.12 (14.70−23.91)
Modi 2022	27.47 (23.22−31.98)
Siena 2021	31.73 (22.25−41.66)
Shitara 2020	42.27 (33.93−50.80)
Cutsen 2021	34.85 (25.18−45.44)
Li 2022	40.53 (31.10−50.24)
Bartsch 2022	23.96 (10.23−42.02)
Tsurutani 2020	28.86 (18.80−40.21)
Modi 2020	16.62 (6.51−30.95)
Modi 2020	32.36 (19.40−47.41)
Tamura 2019	19.16 (12.94−26.50)
Shitara 2019	24.00 (13.85−35.67)
Doi 2017	15.25 (6.15−28.37)
**Overall**	**19.45 (15.70−23.67)**

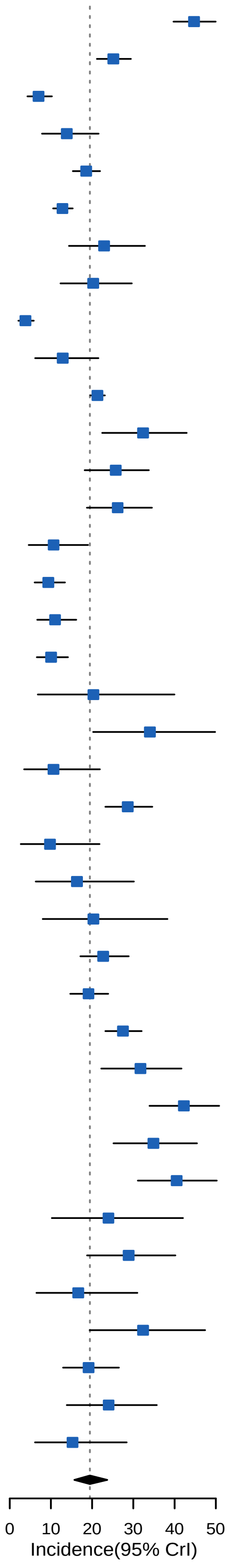

eFigure 4. Overall incidences of adverse events that resulted in drug discontinuation associated with HER2-targeted ADCs (heterogeneity τ = 0.67)

Study	Incidence (95% CrI)
Perez 2019	20.32 (16.35−24.54)
Krop 2017	14.47 (11.29−18.12)
Cortés 2022	5.54 (3.23−8.49)
Cortés 2020	14.49 (8.20−22.27)
Verma 2012	10.04 (7.63−12.68)
Minckwitz 2019	17.78 (15.19−20.58)
Emens 2020	15.08 (8.34−23.34)
Hurvitz 2013	8.17 (3.64−14.55)
Tolaney 2021	17.21 (13.66−21.14)
Thungappa 2022	7.13 (2.70−13.94)
Montemurro 2020	11.85 (10.49−13.26)
Kashiwaba 2015	17.74 (10.63−26.53)
Krop 2011	7.21 (3.58−12.12)
Burris III 2010	5.00 (2.19−9.16)
Gupta 2013	6.37 (2.23−12.95)
WATANABE 2017	3.24 (1.47−5.63)
Krop 2015	2.68 (0.89−5.39)
Yardley 2015	5.73 (3.19−8.89)
Yamamoto 2014	18.83 (6.46−38.56)
Beeram 2012	13.00 (5.17−24.47)
Krop 2010	5.94 (1.46−13.57)
Thuss−Patience 2017	13.64 (9.56−18.30)
Li 2018	6.60 (1.58−15.46)
Iwama 2022	6.09 (1.52−14.48)
Hotta 2017	18.96 (7.20−36.73)
Modi 2020	14.82 (10.23−20.18)
Cortes 2022	13.41 (9.61−17.74)
Modi 2022	15.92 (12.46−19.69)
Siena 2021	9.37 (4.59−15.85)
Shitara 2020	14.66 (9.40−20.97)
Cutsen 2021	14.42 (8.24−22.27)
Li 2022	34.11 (25.30−43.64)
Bartsch 2022	15.60 (5.55−31.27)
Tsurutani 2020	9.10 (3.92−16.15)
Modi 2020	6.28 (1.53−14.44)
Modi 2020	26.98 (14.93−41.56)
Tamura 2019	11.28 (6.52−17.24)
Shitara 2019	12.92 (5.84−22.60)
Doi 2017	9.76 (3.20−20.23)
**Overall**	**10.52 (8.03−13.21)**

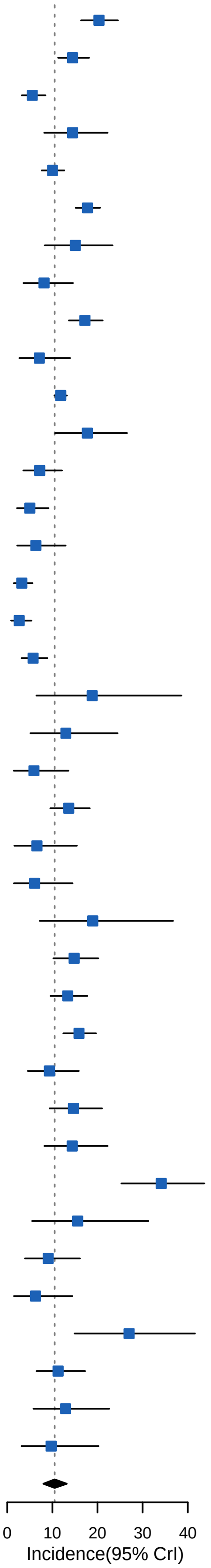

eFigure 5. Overall incidences of fatal adverse events associated with HER2-targeted ADCs (heterogeneity τ = 3.84)

Study	Incidence (95% CrI)
Perez 2019	1.35 (0.47−2.70)
Krop 2017	2.16 (0.99−3.79)
Cortés 2022	0.27 (0.01−1.00)
Cortés 2020	0.57 (0.01−2.41)
Verma 2012	0.85 (0.26−1.78)
Minckwitz 2019	0.23 (0.03−0.66)
Emens 2020	0.62 (0.02−2.65)
Hurvitz 2013	1.46 (0.12−4.76)
Tolaney 2021	0.20 (0.01−0.70)
Thungappa 2022	6.03 (1.69−13.24)
Montemurro 2020	2.23 (1.62−2.92)
Kashiwaba 2015	0.59 (0.02−2.49)
Krop 2011	2.45 (0.57−5.81)
Burris III 2010	0.46 (0.01−1.83)
Gupta 2013	0.72 (0.02−3.12)
WATANABE 2017	0.29 (0.01−1.11)
Krop 2015	0.39 (0.01−1.59)
Yardley 2015	0.30 (0.01−1.12)
Yamamoto 2014	1.46 (0.02−7.63)
Beeram 2012	0.97 (0.02−4.67)
Krop 2010	1.01 (0.02−4.82)
Thuss−Patience 2017	3.36 (1.44−6.04)
Li 2018	1.19 (0.02−5.79)
Iwama 2022	3.16 (0.22−11.52)
Hotta 2017	1.26 (0.02−6.28)
Modi 2020	4.55 (2.09−7.94)
Cortes 2022	0.27 (0.01−1.02)
Modi 2022	3.90 (2.20−6.06)
Siena 2021	10.28 (4.72−17.64)
Shitara 2020	5.84 (2.47−10.78)
Cutsen 2021	0.55 (0.01−2.17)
Li 2022	13.09 (7.09−20.56)
Bartsch 2022	4.10 (0.26−15.32)
Tsurutani 2020	7.16 (2.32−14.55)
Modi 2020	1.13 (0.02−5.56)
Modi 2020	6.83 (1.46−16.61)
Tamura 2019	2.32 (0.54−5.50)
Shitara 2019	3.56 (0.51−9.82)
Doi 2017	1.04 (0.02−5.16)
**Overall**	**1.00 (0.41−1.78)**

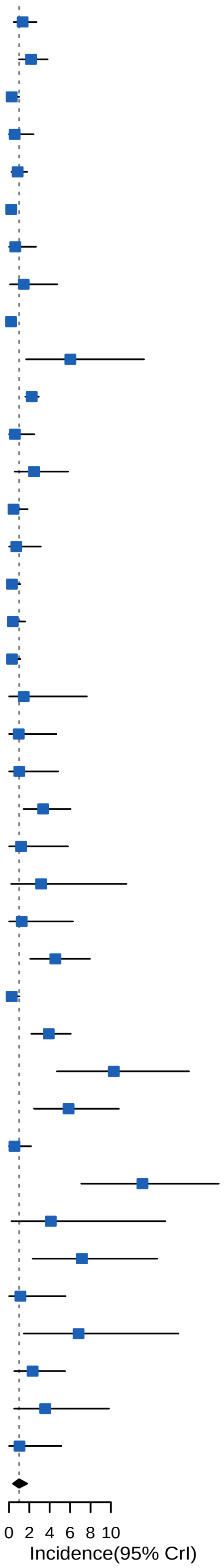

eFigure 6. Funnel plot of the overall incidence of all-grade adverse events (Egger's test: p= 0.136).

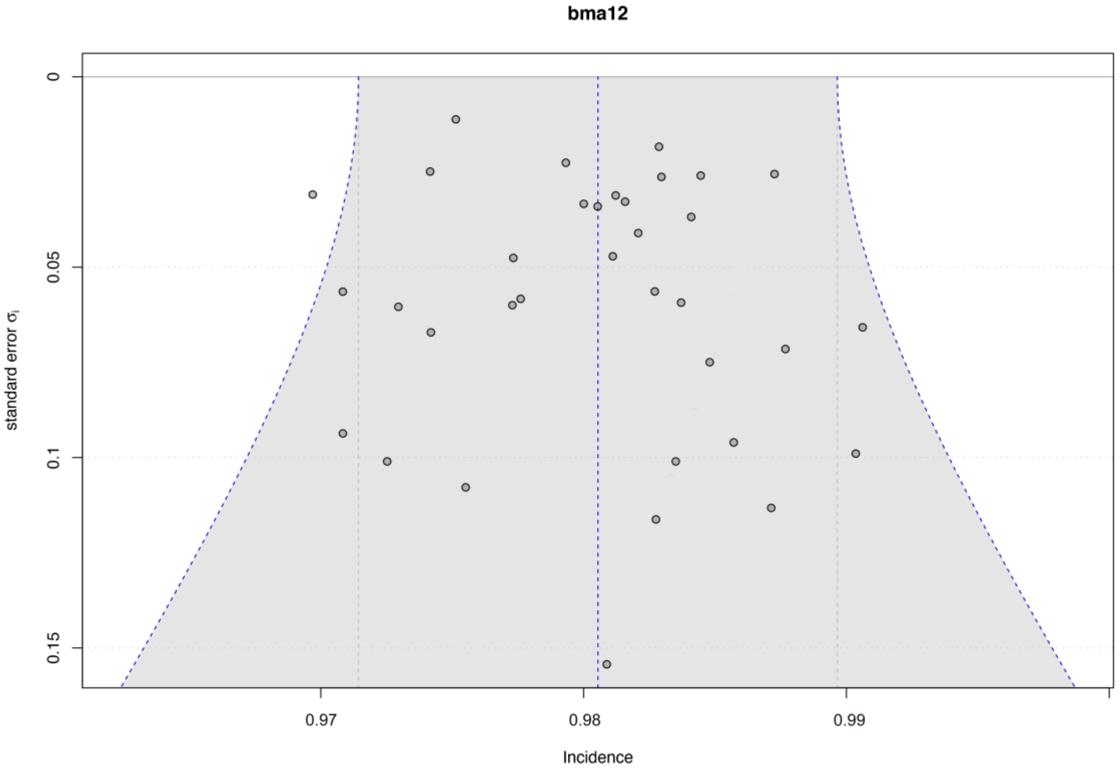

eFigure 7. Funnel plot of the overall incidence of grade 3 or higher adverse events (Egger's test: p= 0.259).

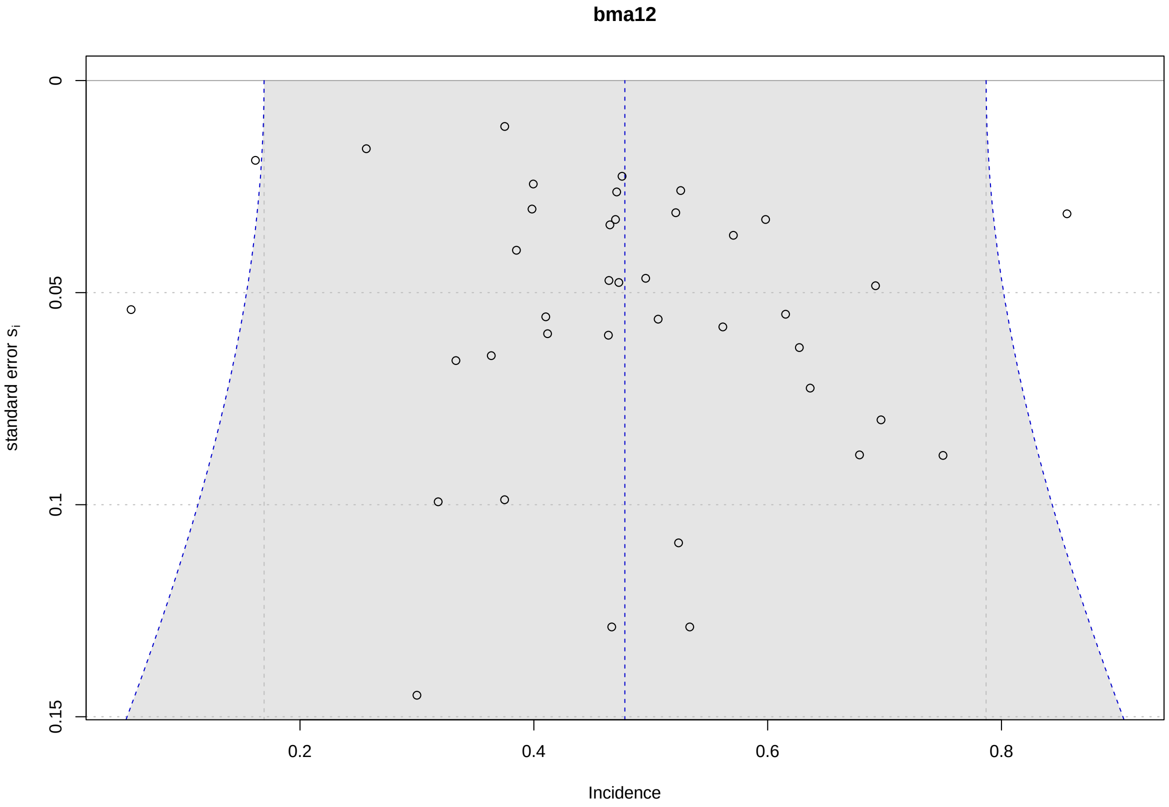

eFigure 8. Funnel plot of the overall incidence of serious adverse events (Egger's test: p= 0.204).

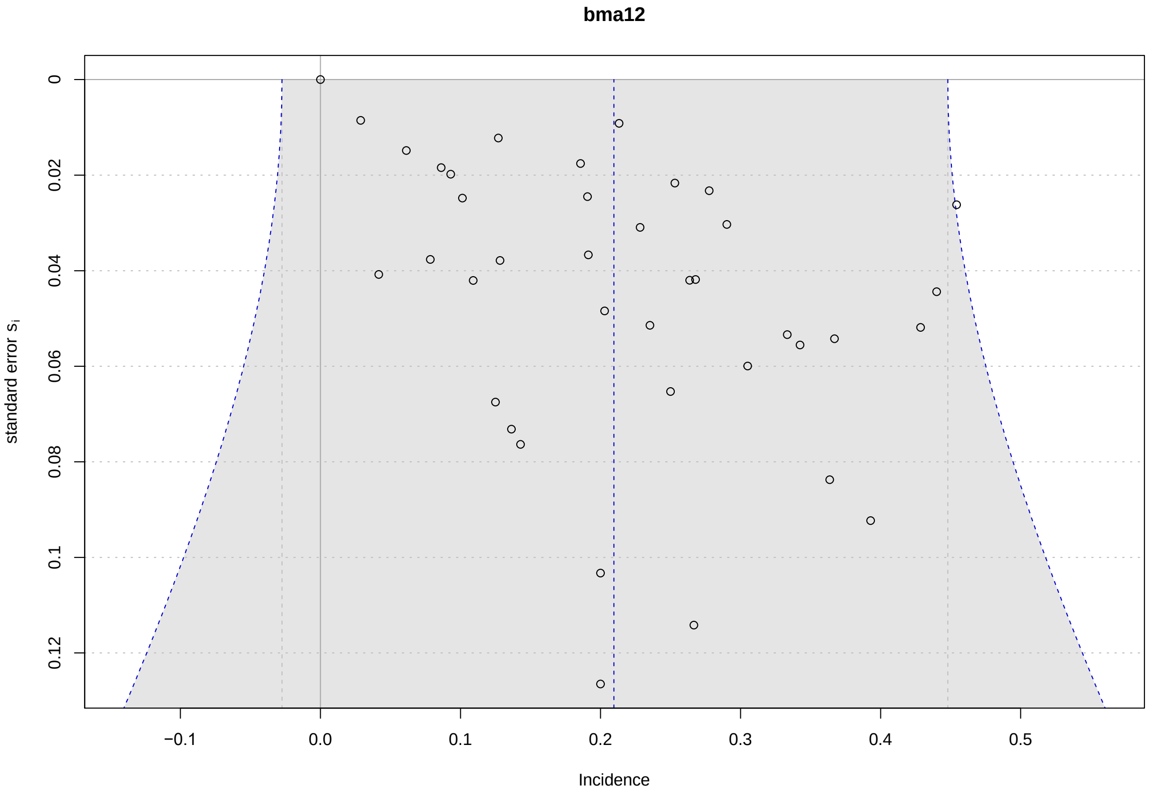

eFigure 9. Funnel plot of the overall incidence of adverse events that resulted in drug discontinuation (Egger's test: p= 0.153).

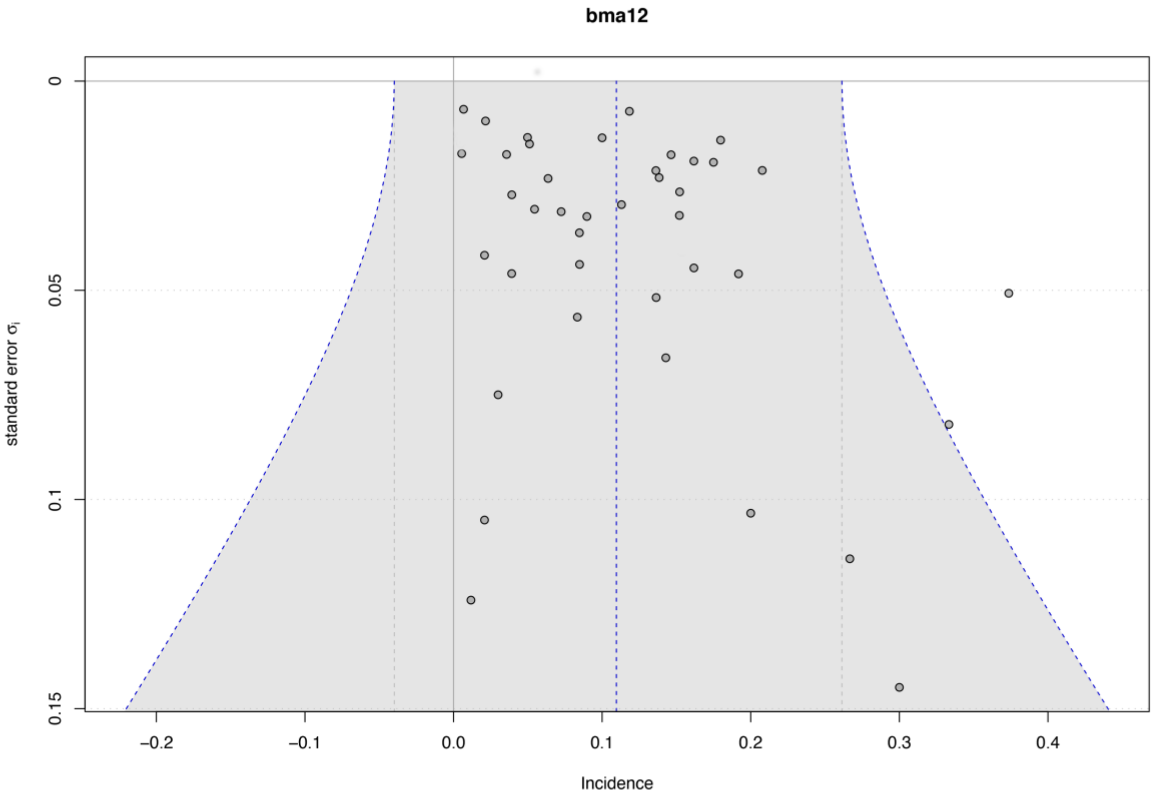

References

1. Liu B, Lahiri P, Kalton G. Hierarchical Bayes modeling of survey-weighted small area proportions. Paper presented at: Proceedings of the American Statistical Association, Survey Research Section2007.
2. Vasudev D, Goswami VR. A Bayesian hierarchical approach to quantifying stakeholder attitudes toward conservation in the presence of reporting error. *Conservation Biology.* 2020;34(2):515-526.
3. Carlin BP, Chib S. Bayesian model choice via Markov chain Monte Carlo methods. *Journal of the Royal Statistical Society: Series B (Methodological).* 1995;57(3):473-484.
4. Sterne JA, Savović J, Page MJ, et al. RoB 2: a revised tool for assessing risk of bias in randomised trials. *bmj.* 2019;366.
5. Slim K, Nini E, Forestier D, Kwiatkowski F, Panis Y, Chipponi J. Methodological index for non-randomized studies (MINORS): development and validation of a new instrument. *ANZ journal of surgery.* 2003;73(9):712-716.
