# Supplementary material for: Treatment-related adverse events associated with HER2-Targeted antibody-drug conjugates in clinical trials: a systematic review and meta-analysis
Source: eClinicalMedicine. 2022 Dec 27;55:101795. doi: 10.1016/j.eclinm.2022.101795 (PMC9874347; doi:10.1016/j.eclinm.2022.101795)
Supplement: Translated Abstract in Chinese [file mmc2.docx]

**The following translations in Chinese were submitted by the authors and we reproduce them as supplied. They have not been peer reviewed. Our editorial processes have only been applied to the original abstract in English, which should serve as reference for this manuscript.**

**摘要**

**背景** 鉴于HER2靶向抗体药物偶联物（ADC）在全球范围内的使用日益增多，总结这些药物的不良反应发生率及其概况对于临床应用至关重要。此次Meta分析旨在综合分析在临床试验中HER2靶向ADC的治疗相关不良事件的平均发生率，并探究不同HER2靶向ADC药物和癌症类型之间是否存在差异。

**方法** 从数据库最早到2022年2月1日，我们在PubMed、Embase、Web of Science和Scopus四个数据库中对相关文献进行了系统搜索，最后一次搜索更新到2022年8月1日。纳入了已发表的针对FDA批准的HER2靶向ADC单药的前瞻性临床试验，其中包含与治疗相关不良事件相关的可用计数数据。主要结局指标是治疗相关不良事件的汇总发生率以及不同药物和癌症类型之间的差异。本研究使用贝叶斯分层建模方法进行数据合成计算，本研究计划在PROSPERO（编号CRD42022331627）中注册。

**结果** 最终分析共包括39项研究（37项试验），涉及五种癌症类型的7688名患者。通过使用贝叶斯分层建模汇总数据，所有级别不良事件、高等级不良事件、严重不良事件和导致停药的不良事件的总体平均发生率分别为98.29%（95%CrI，97.33%-99.07%，τ=1.49）、47.88%（95%CrI，42.74%-53.17%，τ=0.37）、19.45%（95%CrI，15.70%-23.67%，τ=0.55），和10.52%（95%CrI，8.03%-13.21%，τ=0.56）。最常见的所有级别不良事件包括恶心（41.57%；95%CrI，40.46%-42.64%，τ=0.81）、疲劳（35.86%；95%CrI，34.85%-36.96%，τ=0.65）和食欲下降（28.84%；95%CrI，22.93%-36.87%，τ=0.76）。最常见的高等级不良事件包括血小板减少症（8.37%；95%CrI，7.75%-9.07%，τ=0.71）、贫血（6.49%；95%Cr I，5.86%-7.11%，τ=1.06）和中性粒细胞减少症（6.42%；95%CrI，5.76%-7.04%，τ=1.21）。我们发现不同癌症类型以及不同给药方案之间的平均不良事件发生率没有差异。然而，与恩美曲妥珠单抗（T-DM1）相比，德喜曲妥珠单抗（T-DXd）的平均不良事件发生率更高，尤其是对于更高剂量的T-DXd（6.4 mg/kg Q3W）。

**结论** 两种HER2靶向ADC之间的不良事件发生率在不同癌症类型中接近，但不同HER2靶向ADC的不良事件平均发生率有所不同。本研究对于HER2靶向ADC不良事件的全面总结，将对接受HER2靶向ADC治疗的癌症患者及其临床医生使用HER2靶向ADC具有重要意义。

**基金** 中国国家自然科学基金（82073402）和中国湖北省重点研发计划（2020BCA060）资助了本研究。

**关键词** 不良反应；HER2靶向ADC；临床试验；系统综述；贝叶斯meta分析
